# 

**Published:** 1894-09-29

**Authors:** 


### CONTENTS.

**Koch on Cholera** - 507
**Around the Hospitals** - 508
**On Toxic Neutralisations.** — By Sir BENJAMIN WARD RICHARDSON, M.D., F.R.S. I.—Isomeric Variation - 509
**Modern Sociology.**—Labour and Health.—II. Wanted, More Air 510
**Annotations.**—The Diphtheria Problem.—The Effects of Creasote upon Tuberculosis.—The Lighthouse Keepers' Grievance - 511
**Medical Progress and Hospital Clinics:**
Diseases of the Ear. V.—The Artificial Membrana Tympani 513
Notes on the Methods of Performing Paracentesis of the various Cavitives - 514
PROGRESS IN MEDICINE.—Diseases of the Heart and Vascular System - 515
**Notes and News** - 512
**Medical Education in Scotland** - 517
**Medical Education in the Provinces** - 518
**The Institutional Workshop:**
HOSPITAL FINANCE. X.—The Cost of Provisions - 519
REMINISCENCES OF BUDA PESTH AND THE INTERNATIONAL CONGRESS OF HYGIENE - 520
PRACTICAL DEPARTMENTS.—Hospital Laundries (*continued*) - 521
CONSTRUCTION NOTES - 521
PRACTICAL POINTS - 522
**Scraps and Gleanings** - 522
**The Book World of Medicine and Science** - 523
**The Student of Medicine** - 523

### EXTRA SUPPLEMENT.—THE NURSING MIRROR.

**News from the Nursing World.**—Our Christmas Competition—Royal National Pension Fund—Winter Work—Attack on a Matron—In Cash or in Kind?—Sick, but not Certified.—Recommendations.—District Nursing in the Isle of Wight.—Equal Advantages.—An Ambulance Required.—Nurses in Ireland.—A Liberal Frenchman.—For Cancer Patients.—Russian Progress. Our American Sisters.—The Empress of Japan.—Short Items - ccxlv
**Diet in Disease.**—By Mrs. ERNEST HART. IV.—Typhoid Fever - ccxlvii
**Notes from Germany** - ccxlviii
**Notes and Queries** - ccxlix
**The New Laundry Hostel, Guy's Hospital** - ccxlix
**Everybody's Opinion** - cclxix
**Wants and Workers** - ccl
**Nursing in Holland** - ccl

